# Mixed embryonal carcinoma – teratoma cause early puberty: a case report

**DOI:** 10.1186/1687-9856-2015-S1-P93

**Published:** 2015-04-28

**Authors:** Can Thi Bich Ngoc, Vu Chi Dung, Bui Phuong Thao, Nguyen Ngoc Khanh, Tran Ngoc Son, Hoang Ngoc Thach, Nguyen Thi Hoan

**Affiliations:** 1 Department of Endocrinology, Metabolism and Genetics, National Hospital of Pediatrics, Hanoi, Vietnam; 2 Surgical department, National Hospital of Pediatrics, Hanoi, Vietnam; 3 Anatomical department, National Hospital of Pediatrics, Hanoi, Vietnam

**Keywords:** embryonal carcinoma, germ cell tumor

## Introduction

Mixed embryonal carcinoma – teratoma is defined a rare kind of germ cell tumor, especially in children. Objective: a rare case report with early puberty due to mixed embryonal carcinoma – teratoma.

## Subject

A 3 year 7 month old boy presented with early puberty and adrenal tumor in National Hospital of Pediatrics.

## Method

A case report.

## Results

A 3 year 7 month old boy presented with enlargement of penis, acnes, pubic hair, deep voice. He admitted with his height of +2.3SDS, penis length of 6cm, testes volumn of 4ml, pubic hair P2 (tanner). Abdoment ultrasound and CT scan showed adrenal tumor. Serum laboratory showed: 17 OHP 3,8 ng/ml, testosteron 32,3 nmol/l, cortisol 8h 433,9 nmol/l, cortisol 22h 39,2 nmol/l, glucose 3,7 mmol/l, αFP 3945,9 u/l, electrolyte normal; urine VMA 15,5 µmol/24h; bone age 5 years. Surgical showed the tumor did not originate from the adrenal. Histologica showed mixed embryonal carcinoma – teratoma. After surgeon, the signs of virilization reduce, serum testosteron become normal.

## Conclusion

Mixed embryonal carcinoma – teratoma can be caused testosterone excretion and early puberty.

Written informed consent was obtained from the patient's parent or guardian for publication of this Case report (and any accompanying images). A copy of the written consent is available for review by the Editor-in-Chief of this journal.

